# A Low Matrix Effects Analytical Strategy for Diazepam Analysis in Aquatic Products Through Immunomagnetic Beads Purification

**DOI:** 10.3390/foods15132296

**Published:** 2026-06-26

**Authors:** Xiaoyi Lou, Qi Wang, Changling Fang, Xuanyun Huang, Yongfu Shi, Dongmei Huang

**Affiliations:** 1 East China Sea Fisheries Research Institute, Chinese Academy of Fishery Sciences, Shanghai 200090, China; huoxingmayi@126.com (X.L.); 18871385147@163.com (Q.W.); fangcl@ecsf.ac.cn (C.F.); shiyf@ecsf.ac.cn (Y.S.); 2 School of Resources and Environmental Engineering, Shanghai Polytechnic University, Shanghai 201209, China

**Keywords:** immunomagnetic beads, diazepam, UPLC-MS/MS, aquatic products, low matrix effects

## Abstract

A low matrix effects (MEs) analytical strategy was developed for quantifying diazepam (DZP) in aquatic products by combining immunomagnetic bead (IMB)-based sample pretreatment with UPLC-MS/MS. The preparation conditions of IMBs, sample extraction, and purification were systematically optimized. Due to the weak MEs of this method, the quantification of DZP was carried out using a solvent-based calibration curve with an external standard solution. Good linearity was achieved over the range of 0.5–20 ng/mL with a correlation coefficient (R^2^) of 0.9971. The LOD and LOQ were 0.125 μg/kg and 0.25 μg/kg, respectively. Recoveries ranged from 85.5% to 106%, with intra-day and inter-day precisions of 1.16–7.04% and 3.42–8.99%, respectively. This work has established a robust, rapid, and practical methodology for DZP residue monitoring in aquatic products. Moreover, it serves as a methodological foundation for the detection of other low-molecular-weight contaminants in complex food matrices, thereby enhancing the surveillance and risk assessment for aquaculture products.

## 1. Introduction

Diazepam (DZP), also known as Valium, is a benzodiazepine sedative drug widely employed in both veterinary and human clinical medicine owing to its potent sedative, hypnotic, anxiolytic, anticonvulsant, antiepileptic, and central muscle relaxant properties [1,2]. It is classified as a Schedule II psychotropic controlled substance in China due to its toxicity and high risk of dependence and tolerance [3]. Excessive use of DZP could increase liver burden, and disrupt central nervous system function, resulting in adverse symptoms such as irritability, drowsiness, memory impairment, hallucinations, headache, and coma [4,5]. Meanwhile, motor nerve and muscle functions are also suppressed [6].

Recently, DZP has been illicitly employed by certain traders as a fish sedative to reduce fish activity and alleviate stress responses, thereby improving the survival rate of aquatic product transportation [7]. Additionally, DZP has also been incorporated into aquatic feeds as a growth-promoting agent [8]. The illegal use of DZP has posed severe risks to the security and quality of aquatic products [9,10]. In China, DZP has not been authorized for application in aquaculture. As stipulated in the National Food Safety Standard GB 31650-2019 [11], DZP must be absent in all animal-derived foods. Nevertheless, it has been reported in recent years that excessive DZP residue frequently occurs in aquatic products [12,13]. The residues of DZP not only affect the sustainability of the aquaculture industry but also pose possible health risks to consumers. Consequently, developing a rapid and highly sensitive approach for determining DZP in aquatic products is of significant practical importance.

Currently, analytical methods for DZP detection primarily include colloidal gold immunochromatographic assay (CGIA) [14,15], enzyme-linked immunosorbent assay (ELISA) [16,17,18], high-performance liquid chromatography (HPLC) [19,20], gas chromatography-tandem mass spectrometry (GC-MS/MS) [21,22,23], and ultra-performance liquid chromatography-tandem mass spectrometry (UPLC-MS/MS) [24,25,26]. The CGIA and ELISA exhibit poor reproducibility and stability, tend to generate both false positive and false negative results, and cannot achieve accurate quantitative analysis. HPLC exhibits relatively low detection sensitivity and is susceptible to matrix interference during the analysis of drug residues in complex samples [27]. GC-MS/MS requires derivatization reactions, resulting in cumbersome and time-consuming preparation procedures. Owing to its high selectivity, superior sensitivity, and broad applicability, UPLC-MS/MS has been the predominant analytical approach for veterinary drug residue detection [28].

However, the quantitation of DZP in aquatic products by UPLC-MS/MS still presents challenges. Aquatic samples have complex matrices with significant interference from impurities such as proteins, lipids, and fatty acids [29]. Therefore, prior to quantitative analysis, sample pretreatment is required for the extraction and purification of the target analyte to minimize MEs. At present, liquid–liquid extraction (LLE) [30], solid-phase extraction (SPE) [31], and dispersive solid-phase extraction (d-SPE) [8,32] are the dominant sample preparation techniques for analyzing DZP residues in aquatic matrices. LLE exhibits low extraction efficiency and high solvent consumption, posing risks to both the environment and laboratory operators. SPE achieves satisfactory recoveries for spiked samples, yet it involves tedious steps, including preconditioning, loading, washing, and elution, which consume large volumes of solvents and take a long time to operate. Conventional d-SPE absorbents, including octadecylsilyl (C18) and primary secondary amine (PSA), are unable to completely remove matrix interferences, leading to significant MEs that compromise the quantitative accuracy of the UPLC-MS/MS method [33]. Therefore, the development of convenient and efficient sample pretreatment procedures is pivotal for enhancing the detection efficiency of the UPLC-MS/MS method.

In recent years, immunomagnetic beads (IMBs), as a kind of adsorbent functionalized with antibodies against the target, have received significant attention in the scope of sample pretreatment [34,35]. IMBs are synthesized by conjugating antibodies onto the surface-functionalized magnetite Fe_3_O_4_ nanoparticles. In the presence of an external magnetic field, IMBs enable rapid directional manipulation and efficient isolation, concentration, and transport of target compounds. Owing to its operational simplicity, high purification efficiency, and superior specificity, this approach exhibits considerable potential for detecting small-molecule compounds in complex matrices. To date, a variety of IMB-based sample pretreatment protocols have been proposed to analyze the target compounds in complex sample matrices [36,37,38,39].

In this study, IMBs were prepared through specific antigen–antibody interactions to capture DZP and subsequently applied to the extraction and purification of DZP from various aquatic product matrices. Compared with conventional sample pretreatment methods, the proposed approach was easy to operate, could improve pretreatment efficiency, and reduce reagent consumption, which enabled rapid sample purification. Quantitative analysis could be directly performed using the solvent-based standard curve, due to the low MEs of this method. Coupling with UPLC-MS/MS, a robust and highly specific analytical approach was established for quantitative analysis of DZP residues in aquatic products. The objective of this research is to investigate the potential of IMBs in determining DZP residues in aquatic products, thereby optimizing sample pretreatment procedures and providing technical reference for other target determinations in complex matrices.

## 2. Materials and Methods

### 2.1. Chemicals and Reagents

DZP standard (purity 99.9%) was purchased from Tanmo Quality Inspection Standard Material Center. Magnetic beads (MBs, Carboxyl magnetic beads^®^ Magrose NHS) supplied by Beaver Biotechnology (Suzhou, China) were used for IMBs synthesis. DZP monoclonal antibody was obtained from Shanghai Bainong Biotechnology. Acetonitrile (ACN), methanol (MeOH), ethyl acetate (EtOAc), and fomic acid (HPLC grade) were purchased from Sinopharm Chemical Reagent Co., Ltd. (Shanghai, China). The BCA protein Assay kit was from Beyotime Biotechnology (Shanghai, China). Ultrapure water was obtained from Milli-Q (Millipore, Bedford, MA, USA).

### 2.2. Instruments

An ACQUITY UPLC I-Class system equipped with a Xevo TQ-XS mass spectrometer (Waters, Milford, CT, USA) was used for UPLC/MS/MS analyses. Sample mixing was performed with an MS3D vortex mixer (IKA, Staufen, Germany). The ultrasonic treatment was conducted by a KQ-300E ultrasonic processor (Kunshan Ultrasonic Instrument Co., Ltd., Suzhou, China). Multifuge X3R centrifuge (Thermo Fisher Scientific, Waltham, MA, USA) and Rotavapor R210 (Buchi Instruments & Equipment Co., Ltd., Shanghai, China) were used during sample preparation. Scanning electron microscope (SEM, ZEISS Sigma 300, Jena, Germany) and transmission electron microscope (TEM, JEOL JEM-2100F, Tokyo, Japan) were employed to observe the morphology and structure of IMBs. The magnetic property of IMBs was evaluated via a vibrating sample magnetometer (VSM, LakeShore 7404, O’Fallon, MO, USA).

### 2.3. Preparation of IMBs

MBs were thoroughly vortexed and mixed, then subjected to magnetic separation to remove the supernatant. Pre-cooled 1 mmol/L HCl was added to wash the beads to prevent hydrolysis of NHS groups. After magnetic separation and supernatant removal, an appropriate amount of 2 mg/mL DZP monoclonal antibody was added and followed by a 2 h incubation at ambient temperature. After the conjugation, the unbound antibody in the supernatant was discarded by magnetic separation. Blocking was performed with 3 mol/L ethanolamine at 4 °C overnight. Subsequently, the IMBs were washed three times using 0.01 mol/L phosphate-buffered solution (PBS, pH 7.4) and re-suspended in PBS for storage at 4 °C. The BCA Protein Assay Kit was employed to detect the protein concentration in the supernatant after conjugation. The effects of antibody dosage, conjugation pH, and conjugation time on antibody conjugation efficiency were investigated, aiming to obtain the optimal preparation conditions for IMBs. The antibody coupling amount (A) and coupling rate (R%) on the bead surface were used to evaluate antibody conjugation efficiency, which was calculated as follows:

(1) $$A=m_{0}-m_{1}$$

(2) $$R\%=\frac{A}{m_{0}}\times 100\%$$

where *m*_0_ is the amount of initial added antibody and *m*_1_ is the remaining antibody amount in supernatant [40].

### 2.4. Sample Preparation and Extraction

Grass carp (*Ctenopharyngodon idella*), crucian carp (*Carassius auratus*), large yellow croaker (*Larimichthys crocea*), Pacific white shrimp (*Litopenaeus vannamei*), and Chinese mitten crab (*Eriocheir sinensis*) were purchased from local agricultural markets in Shanghai, China. Shells, viscera, and bones were discarded, and the edible parts of the samples were homogenized and stored at −20 °C until analysis. An accurately weighed 2.0 g of sample was placed in a 50 mL polypropylene tube. Next, 10 mL of 1% ammoniated acetonitrile (*v*/*v*) was added, followed by vigorous shaking for 2 min and ultrasonic treatment for 5 min. Subsequently, the mixture was centrifuged at 4000 rpm for 5 min to collect the supernatant and then concentrated to dryness via rotary evaporation at 45 °C. The obtained dry extract was diluted with 1 mL of ultrapure water for further purification.

### 2.5. Purification Based on IMBs

One milligram of IMBs was introduced into the solution for purification. After vortex mixing for 5 min for DZP adsorption, the supernatant was removed by magnetic separation. The elution was performed by adding 500 μL of methanol, followed by another 5 min vortex step. Magnetic separation was performed to obtain the supernatant, which was then subjected to a 0.22 μm polytetrafluoroethylene membrane and subsequently detected with UPLC-MS/MS.

### 2.6. UPLC-MS/MS Analysis Method

An ACQUITY UPLC BEH C18 column (1.7 μm; 50 × 2.1 mm) (Waters, Milford, CT, USA) was employed for chromatographic separation. The column was kept at 30 °C, and a 5 μL aliquot of the sample was injected at a flow rate of 0.3 mL/min. The mobile phase comprised 0.1% formic acid in water (phase A) and methanol (phase B). The elution procedure was as below: 0–0.5 min, 90% A; 0.5–1.5 min, linearly reduced from 90% A to 50% A; 1.5–2.0 min, 50% A; 2.0–3.0 min, linear gradient from 50% A to 5% A; 3.0–6.0 min, 5% A; 6.0–7.0 min, switched instantaneously from 5% A to 90% A and held. The positive electrospray ionization (ESI^+^) and multiple reaction monitoring (MRM) mode were employed in mass spectrometry. The instrument parameters were set as follows: the source temperature was 150 °C, the capillary voltage was 3.0 kV, the cone gas flow rate was 150 L/h, the collision gas flow rate was 0.14 mL/min, the desolvation temperature was 500 °C, and the desolvation gas flow rate was 800 L/hr. The quantitative ion pairs were 285.1 ⟶ 193.1, and the qualitative ion pairs were 285.1 ⟶ 154.0, with both collision energies 26 eV and cone 30 V.

### 2.7. Method Validation

The validation of the method was conducted in accordance with European Union Commission Decision 2002/657/EC [41]. Specificity, linearity, the limit of detection (LOD), the limit of quantification (LOQ), MEs, accuracy, and precision were assessed. Specificity was assessed by analyzing negative samples and examining the chromatographic profile for any interference signals within the retention time window. The linearity was evaluated by the calibration curves prepared using methanol with the concentration range of 0.5–20 ng/mL for DZP. Quantitative analysis was carried out by constructing a calibration curve, where peak areas were plotted against respective DZP concentrations. The LOD and LOQ were estimated by spiking DZP into blank matrices, with signal-to-noise ratios (S/N) of 3 and 10. The accuracy and precision were investigated by measuring recoveries, inter-day relative standard deviations (RSDs), and inter-day RSDs for six independent replicates at three different spiked concentration levels (1, 5, and 10 times LOQ). The ME was assessed through making a comparison between the slopes of solvent-based standard curves versus those obtained with matrix-matched standard curves. The formula was shown as follows [42]:

(3) $$Matrix\;effect\left(\%\right)=\frac{{Slope}_{matrix}-{Slope}_{solvent}}{{Slope}_{solvent}}\times 100$$

According to the evaluation criteria, ME is considered weak when the value is ranged in ±20%. Moderate ME corresponds to values from −50% to −20% or 20% to 50%. Strong ME is defined when the values are beyond ±50%.

## 3. Results and Discussion

### 3.1. Characterization of IMBs

**Figure 1:**
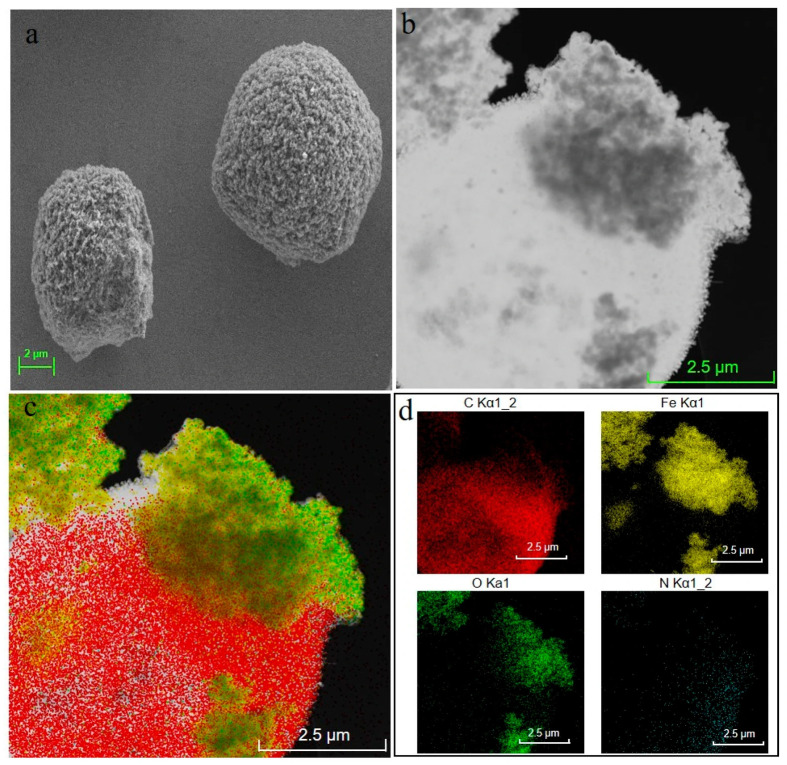
Morphology of IMBs. (**a**) SEM image of IMBs. (**b**) TEM image of IMBs. (**c**,**d**) Elemental mapping of IMBs.

SEM and TEM were employed to characterize the surface and microscopic morphology of IMBs and MBs (Figure 1 and Figure S1). The SEM image showed that IMBs and MBs both presented spherical morphology with a rough granular surface, and the average particle size was approximately 10 μm (Figure 1a and Figure S1a). The antibody coupling process did not significantly change the size of the particles. In contrast to MBs, the IMBs exhibited superior dispersibility, which could be attributed to the immobilization of antibodies effectively attenuating magnetic interactions and thus alleviating agglomeration. The TEM image revealed that crowded Fe_3_O_4_ nanoparticles (the dark zone) were wrapped by agarose gel (the gray area) (Figure 1b). Elemental mapping demonstrated that the IMBs contained C, N, O and Fe elements (Figure 1c,d). Compared with the elemental mapping of MBs (Figure S1c,d), the percentage content of C increased significantly from 22.99% to 80.31%, while the percentage content of Fe decreased from 74.33% to 10.96%. These variations in elemental contents proved that antibodies were coupled on the MBs, and the IMBs were successfully prepared. The atomic percentage contents of C, N, O and Fe in IMBs and MBs were listed in Table S1.

**Figure 2:**
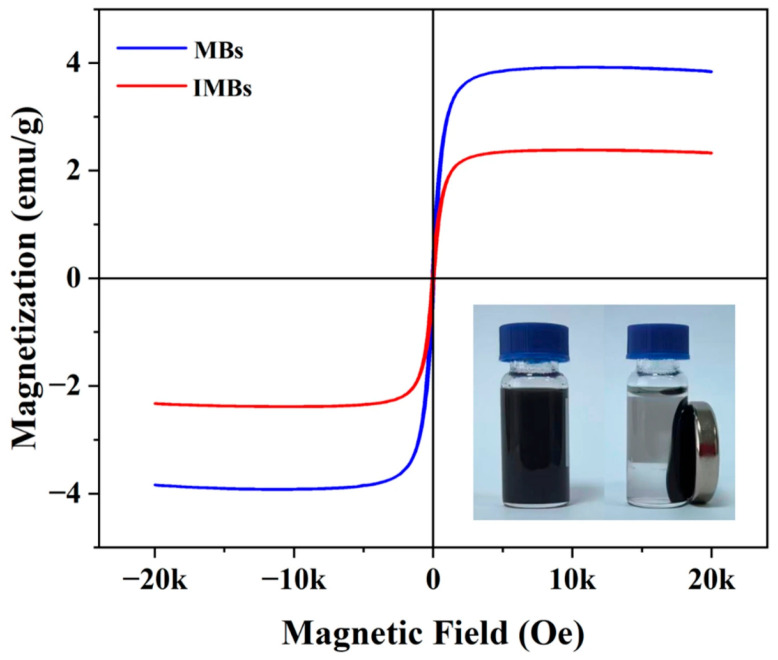
Magnetization curve of MBs and IMBs.

The VSM was used to characterize the magnetic property of IMBs and MBs. As demonstrated in Figure 2, in the absence of an external magnetic field (0 Oe), both IMBs and MBs exhibited negligible remanent magnetization and coercive fields, which are consistent with superparamagnetic behavior and the lack of magnetic hysteresis. The saturation magnetic strength values of MBs and IMBs were 3.92 emu/g and 2.38 emu/g, respectively. The decline in saturation magnetization was ascribed to the introduction of nonmagnetic antibody layers on the MBs surface, providing additional evidence for the effective surface functionalization of the MBs. However, the present magnetism of IMBs was still strong enough to be separated from sample solutions within 10 s via a magnet (inset in Figure 2).

### 3.2. Optimization of IMBs

**Figure 3:**
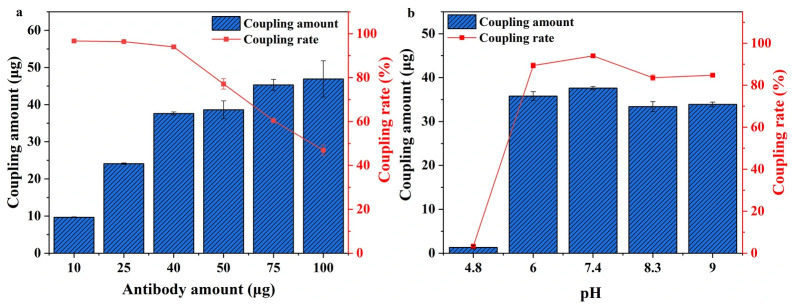
Optimization of IMBs coupling conditions: (**a**) Coupling amount and coupling rate with different antibody amount; (**b**) coupling amount and coupling rate in 0.1 mol/L PBS with different pH. Data were presented as mean ± standard deviation (SD) of three independent replicates.

This study systematically investigated the effects of antibody amount, coupling pH, and coupling time on the coupling efficiency. DZP monoclonal antibodies at doses of 10, 25, 40, 50, 75, and 100 μg were separately coupled with 1 mg of MBs, and the results were shown in Figure 3a. As the antibody dosage increased, the amounts of antibody coupled to the MBs rose progressively, whereas the coupling rate declined gradually, suggesting that the binding sites on the MBs were approaching saturation. To avoid antibody waste, 40 μg antibody per mg beads was chosen for further optimization studies.

The conjugation efficiency between amino groups of antibodies and carboxyl groups on MBs is strongly influenced by the pH of the reaction system, as excessively acidic or alkaline conditions can impair antibody activity [43]. Coupling experiments were performed in five different 0.1 mol/L PBS solutions with pH values of 4.8, 6.0, 7.4, 8.3, and 9.0 to determine the optimal coupling pH. The results were shown in Figure 3b. At pH 4.8, both coupling efficiency and amount were the lowest (only 3.33%), which may be attributed to the coupling pH being lower than the isoelectric point of the antibody, leading to low covalent coupling efficiency. At pH 7.4, the coupling rate and amount reached a maximum of 94.0%.

The influence of the coupling time between antibodies and magnetic beads was shown in Figure S2. The coupling efficiency and amount gradually increased with prolonged coupling time. When the coupling time ranged from 60 to 90 min, the coupling efficiency reached 94.0–94.5%, and the growth trend tended to be stable. Considering both detection performance and time cost, the optimum coupling time was identified as 60 min. Therefore, the final optimal coupling conditions were established as follows: 40 μg of DZP monoclonal antibody per mg of magnetic beads, pH 7.4, and a coupling time of 60 min, with a coupling efficiency of approximately 94.0%.

### 3.3. Sample Extraction

The influence of different extractants on the extraction efficiency of DZP was first investigated. Negative matrix samples (2.0 g) were spiked with 10 ng DZP and extracted with 10 mL of various solvents, including methanol, 1% formic acid methanol (*v*/*v*), 1% ammonia methanol (*v*/*v*), acetonitrile, 1% formic acid acetonitrile (*v*/*v*), and 1% ammonia acetonitrile (*v*/*v*). The extracted solution was directly injected into LC-MS/MS for quantification to determine DZP extraction efficiency. As shown in Figure S3, the findings revealed that both 1% formic acid methanol and 1% formic acid acetonitrile exhibited unsatisfactory DZP extraction performance, with corresponding recoveries lower than 62.2%. In contrast, 1% ammoniated acetonitrile exhibited optimal extraction performance, with recovery rates ranging from 93.7% to 96.7% across five tested matrices. This enhanced efficiency could be attributed to the fact that alkaline conditions can inhibit the protonation of DZP, facilitating the formation of its neutral molecular form, which is more readily soluble in organic solvents [44]. Thus, 1% ammoniated acetonitrile was ultimately chosen as the most effective extractant for DZP in the following experiments.

### 3.4. Purification Based on IMBs

**Figure 4:**
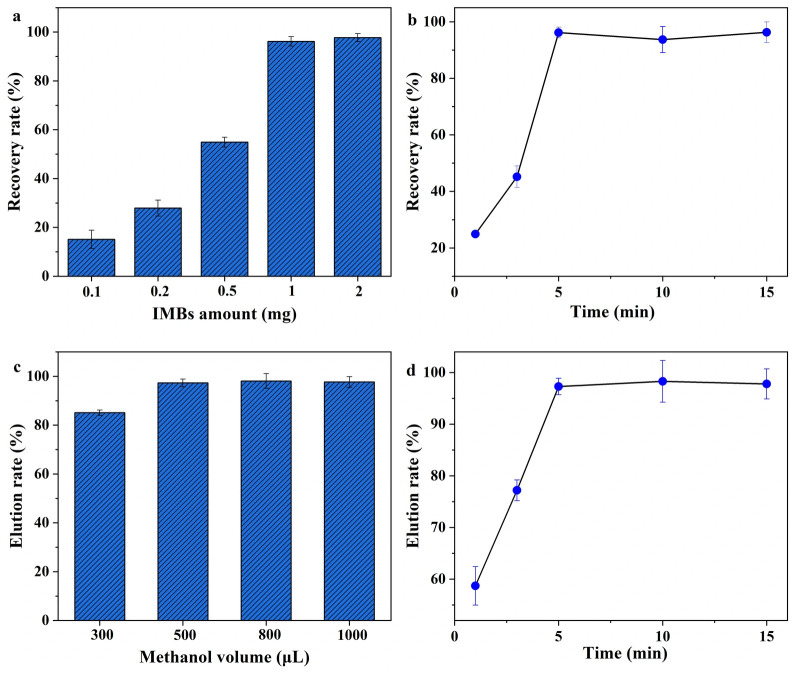
Optimization of purification conditions based on IMBs: (**a**) DZP recovery rate with different amounts of IMBs. (**b**) DZP recovery rate with different adsorption times. (**c**) Elution rate with different methanol volumes. (**d**) Elution rate with different elution times. Data were presented as mean ± standard deviation (SD) of three independent replicates.

To study the effect of IMB’s amount on the adsorption efficiency of DZP, 40 ng of DZP was spiked into 1 mL of the ultrapure water. Subsequently, different IMB dosages of 0.1, 0.2, 0.5, 1, and 2 mg were separately added. The DZP adsorption rate was determined to optimize the IMBs dosage, and the results were given in Figure 4a. The adsorption efficiency of DZP increased with the rising dosage of IMBs, indicating rapid binding interaction between the beads and DZP. The highest adsorption rate of 96.2% was achieved with 1 mg IMBs. As the IMBs dosage further increased, the adsorption rate plateaued. To reduce sample pretreatment time, the adsorption duration was further optimized. Adsorption experiments were conducted by using 1 mg of IMBs to capture 40 ng of DZP at time intervals of 1, 3, 5, 10, and 15 min, respectively (Figure 4b). The results illustrated that the prolonged adsorption duration led to a progressive enhancement in DZP adsorption efficiency, and it reached the maximum when the adsorption time was 5 min. Thus, the optimal dosage of IMBs was 1 mg with the adsorption duration of 5 min.

Methanol was employed as the eluent solvent for the IMBs, and the effects of eluent volume and elution time on the elution efficiency were systematically evaluated (Figure 4c,d). The results demonstrated that when methanol volumes ranging from 300 μL to 1000 μL were used, the elution rate gradually increased from 85.1% to 98.1%. Nevertheless, the elution rate remained stable when the methanol volume exceeded 500 μL. To minimize the consumption of organic solvents, 500 μL of methanol was ultimately selected as the optimal eluent volume. In addition, the elution rate increased progressively as the elution time extended, peaking at 97.3% when the elution time was set at 5 min. Further prolongation of the elution time did not significantly improve the elution efficiency. Thus, 5 min was identified as the optimal elution time.

### 3.5. Method Validation

#### 3.5.1. Specificity

Figure S4 illustrates the MRM chromatogram obtained for 1 ng/mL DZP standard solution under the optimized UPLC-MS/MS conditions. The MRM chromatograms of five blank aquatic samples are presented in Figures S5–S9, respectively. The results demonstrated that DZP exhibited a sharp and symmetric peak with a high mass spectrometric response intensity at 3.28 min. No obvious interfering signals appeared within the relevant retention window, indicating that the method developed in this study displayed excellent specificity.

#### 3.5.2. Matrix Effects

**Figure 5:**
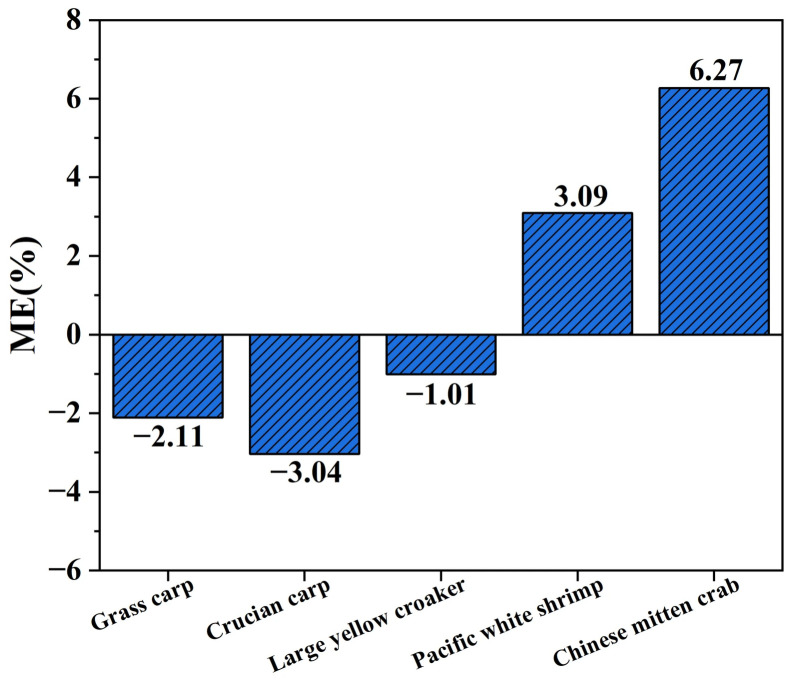
Matrix effects of DZP in various aquatic products.

Impurities that are not fully removed during sample preparation may induce MEs. In mass spectrometry analysis, co-elution of impurities can affect the ionization efficiency, causing signal enhancement or suppression [45]. Consequently, the quantitative accuracy of the analytical targets may be compromised. To evaluate MEs, this study analyzed the MEs in five aquatic products through the comparison of the calibration curve slopes obtained from matrix-matched standards and those derived from solvent-based standards. As presented in Figure 5, matrix inhibition effects were observed in grass carp, crucian carp, and large yellow croaker samples, while matrix enhancement effects occurred in Pacific white shrimp and Chinese mitten crab. Fish and crustacean samples exhibit different matrix effects in LC-MS/MS, likely due to compositional differences in phospholipids, polar lipids, proteins, and amino acids that modulate ionization efficiency in the electrospray ionization process. However, all measured ME values of DZP in these five aquatic products ranged from −3.04% to 6.27%, indicating the MEs were weak and could be considered negligible. These results suggested that the sample pretreatment procedure exhibited satisfactory purification efficiency, significantly minimizing matrix interference and, thus, the solvent calibration curve can be directly used for quantitative analysis.

#### 3.5.3. Linearity, LOD, and LOQ

DZP standard working solutions at concentrations of 0.5, 1.0, 2.0, 5.0, 10, and 20 ng/mL were prepared using methanol as the solvent. A standard curve was established by plotting the peak areas obtained from mass spectrometric detection (Y-axis) against the corresponding DZP concentrations (X-axis). The linear equation and correlation coefficient (R^2^) were obtained via linear regression analysis. The results showed that the linear regression equation was y = 32979x + 8754.93 with an R2 of 0.9971, indicating that DZP exhibited favorable linearity within the concentration of 0.5–20 ng/mL. The LODs and LOQs were determined based on the chromatographic peaks of blank spiked samples, with concentrations corresponding to S/N of 3 and 10, respectively. These findings demonstrated that the LOD and the LOQ of DZP in all five aquatic matrices were 0.125 μg/kg and 0.25 μg/kg, respectively.

#### 3.5.4. Accuracy and Precision

To assess the accuracy and precision of the proposed approach, DZP was spiked into five kinds of negative aquatic matrices at three concentration levels of 0.25,1.25 and 2.5 μg/kg (1, 5, and 10 times LOQ), respectively. The recoveries were calculated by comparing the measured concentration of processed samples with the true spiked concentration. Intra-day and inter-day precision were determined as the RSD from six replicate analyses at the three concentration levels within one day and over three consecutive days, respectively. As given in Table 1, the recoveries for DZP across the three spiking levels ranged from 85.5% to 106%, with intra-day RSDs and inter-day RSDs ranging from 1.16% to 7.04% and 3.42% to 8.99%, respectively. Recovery rates across all tested matrices fell within the range of 70% to 120%, and the relative standard deviations (RSDs) were all below 20%. These findings indicated that the proposed approach exhibited favorable recovery rates, high sensitivity, and satisfactory reproducibility, making it applicable for the measurement of DZP in diverse aquatic products, such as fish, shrimp, and crabs.

### 3.6. Real Sample Analysis

To explore the practical feasibility of the developed approach in real samples, a total of 30 aquatic products (including grass carp, crucian carp, common carp, silver carp, bighead carp, bream, large yellow croaker, Pacific white shrimp, and Chinese mitten crab) were obtained from several aquatic products markets in Shanghai, China. The results revealed that DZP was found in one bream sample with 0.68 ± 0.07 μg/kg. For verification, these samples were also analyzed following the Chinese Entry-Exit Inspection and Quarantine Industry Standard (SN/T 3235-2012) [46], yielding a comparable result of 0.61 ± 0.04 μg/kg. This further confirmed the favorable analytical performance of the established method.

### 3.7. Methods Comparison

A thorough evaluation was carried out to contrast the proposed approach against existing techniques reported in the literature for DZP detection in aquatic products, including instrument, pretreatment, quantification, LOD, LOQ, recovery, precision, and matrix (Table 2). Compared with existing methods, the proposed method offered several distinct advantages. Firstly, the proposed method employed IMBs for sample pretreatment, relying on the mechanism of specific antigen–antibody immunoreaction, which could only selectively capture trace diazepam molecules in aquatic products. This biological affinity mechanism is an intrinsically different design from the traditional chemical adsorption, representing a novel target-specific pretreatment strategy for DZP detection in aquatic products. This present process exhibited high specificity, satisfactory recovery, and superior sensitivity, along with lower LOD and LOQ. These advantages render it distinctly superior in the monitoring of trace DZP residues. Additionally, the weak MEs of this method enabled satisfactory quantification using solvent-based external calibration curves, thereby avoiding the use of matrix-matched calibration or costly deuterated internal standards, which greatly decrease the detection costs. Most traditional methods utilize SPE cartridges for sample pretreatment, involving complex operations such as conditioning, loading, washing, and elution with high consumption of organic reagents. In contrast, the proposed method was simpler to operate with reduced organic solvent usage (e.g., only 10 mL per extraction), which was conforming to the principles of green chemistry. In summary, the method established herein was characterized by simplicity, rapidity, stability, and high sensitivity, making it applicable for the detection of DZP residues in aquatic products.

## 4. Conclusions

This research established a low MEs analytical approach combining IMBs purification with UPLC-MS/MS for detecting DZP residues in aquatic products. IMBs were prepared using MBs and the DZP monoclonal antibody. The antibody amount, coupling pH, and coupling time were investigated to achieve optimal IMBs. The IMBs exhibited strong magnetic responsiveness, high specificity, and efficient purification capability for complex samples. The current method also involved systematic optimization of both the extraction solvent and the purification parameters of IMBs. The validated approach exhibited satisfactory accuracy and precision and could be effectively utilized for detecting DZP in real aquatic products. This research provided a novel, simple, and efficient analytical strategy with low MEs for quantitative analysis of DZP residues in aquatic products, which demonstrated significant promise for application in food security monitoring.

## Figures and Tables

**Table 1:** Recovery and precision of DZP in spiked samples of five aquatic product matrices (*n* = 6).

Matrix	Spiked Amount (μg/kg)	Recovery (%)	Intra-Day RSD (%)	Inter-Day RSD (%)
grass carp	0.25	89.7	6.03	8.99
	1.25	88.6	2.68	6.57
	2.5	85.5	4.58	6.04
crucian carp	0.25	90.3	5.63	5.21
	1.25	91.6	4.72	7.72
	2.5	85.5	2.39	8.82
large yellow croaker	0.25	92.0	3.36	6.38
	1.25	90.5	1.16	6.56
	2.5	89.3	7.04	8.42
Pacific white shrimp	0.25	94.7	5.09	6.56
	1.25	89.0	5.20	4.98
	2.5	97.7	5.28	5.31
Chinese mitten crab	0.25	106	2.30	6.68
	1.25	93.5	1.22	3.42
	2.5	87.1	5.57	5.75

**Table 2:** Comparison of the proposed method with other reported methods for DZP analysis in aquatic products.

Instrument	Pretreatment	Quantification	LOD (μg/kg)	LOQ (μg/kg)	Recovery (%)	Precision (%)	Matrix	Ref.
HPLC-MS/MS	QuEChERS (PSA, MWCNTs)	Matrix-match calibration curves	0.5	2.5	96.0–108.8	<10	Carp	[32]
UPLC-MS/MS	QuEChERS (C18)	Matrix-matched calibration curve with internal standard correction	0.53	1.76	96.1–97.6	2.5–6.2	Freshwater fish	[8]
UPLC-MS/MS	SPE (Florisil, C18, PSA, NH2)	Solvent-based calibration curve with internal standard correction	0.03–0.08	0.10–0.24	81.6–113	2.0–5.6	Carps, grass carp, Hypophthalmichthys nobilis, tilapia, crucian carp, turbot, shrimp, Hypophthalmichthys molitrix, catfish and mussel	[26]
UPLC-MS/MS	QuEChERS (Florisil, C18)	Matrix-matched calibration curve with internal standard correction	/	0.1	89.8–97.2	1.3–9.3	Grass carp, tilapia, crucian, silver carp, bighead carp	[47]
UPLC-QEOrbitrap-MS and UPLC-MS/MS	SPE (MCX)	Solvent-based calibration curve with internal standard correction	0.1	0.2	78.3–111.6	2.42–11.61	Large yellow croaker, Pacific white shrimp, bay scallop, three-wart crab, and squid	[48]
UPLC-MS/MS	QuEChERS (Na_2_SO_4_, C18)	Matrix-matched calibration curve	0.04	0.13	72.42–87.13	5.13–8.68	aquatic products	[49]
UPLC-MS/MS	MSPE * (Fe_3_O_4_@SiO_2_-PSA, C18)	Matrix-matched calibration curve	0.2	0.5	74.9–109	1.24–11.6	Carassius auratus, Litopenaeus vannamei, Portunus trituberculatus, and Mytilus edulis	[44]
UPLC-MS/MS	MSPE (Fe_3_O_4_@SiO_2_-DVB-NVP)	Solvent-based calibration curve	0.2	0.5	89.3–119.7	0.8–10.2	Carassius auratus, Litopenaeus vannamei, Portunus trituberculatus, and Mytilus galloprovincialis	[50]
UPLC-MS/MS	MSPE (IMBs)	Solvent-based calibration curve	0.125	0.25	85.5–106	1.16–8.99	grass carp, crucian carp and large yellow croaker, Pacific white shrimp and Chinese mitten crab	Present work

* MSPE: Magnetic Solid Phase Extraction.

## Data Availability

The original contributions presented in the study are included in the article/Supplementary Materials; further inquiries can be directed to the corresponding authors.

## Supplementary Materials

The following supporting information can be downloaded at: https://www.mdpi.com/article/10.3390/foods15132296/s1. Figure S1: Morphology of MBs. (a) SEM image of MBs. (b) TEM image of MBs. (c,d) Elemental mapping of MBs; Figure S2: Coupling amount and coupling rate with different coupling times. Data were represented as mean ± standard deviation (SD) of three replicates; Figure S3: DZP recovery rate with different extraction solutions. Data were represented as mean ± standard deviation (SD) of three replicates; Figure S4: The MRM chromatogram of DZP standard solution (1 ng/mL); Figure S5: The MRM chromatogram of DZP in a blank grass carp matrix; Figure S6: The MRM chromatogram of DZP in a blank crucian carp matrix; Figure S7: The MRM chromatogram of DZP in a blank large yellow croaker matrix; Figure S8: The MRM chromatogram of DZP in a blank Pacific white shrimp matrix; Figure S9: The MRM chromatogram of DZP in a blank Chinese mitten crab matrix; Table S1: Atomic percentage contents of IMBs and MBs.
