# Supplementary material for: A Low Matrix Effects Analytical Strategy for Diazepam Analysis in Aquatic Products Through Immunomagnetic Beads Purification
Source: Foods. 2026 Jun 26;15(13):2296. doi: 10.3390/foods15132296 (PMC13360824; doi:10.3390/foods15132296)
Supplement: Supplementary file 1 [file foods-15-02296-s001.zip › foods-4384325-supplementary.pdf]

Supplementary Material

A low matrix effects analytical strategy for diazepam analysis in aquatic products through immunomagnetic beads purification

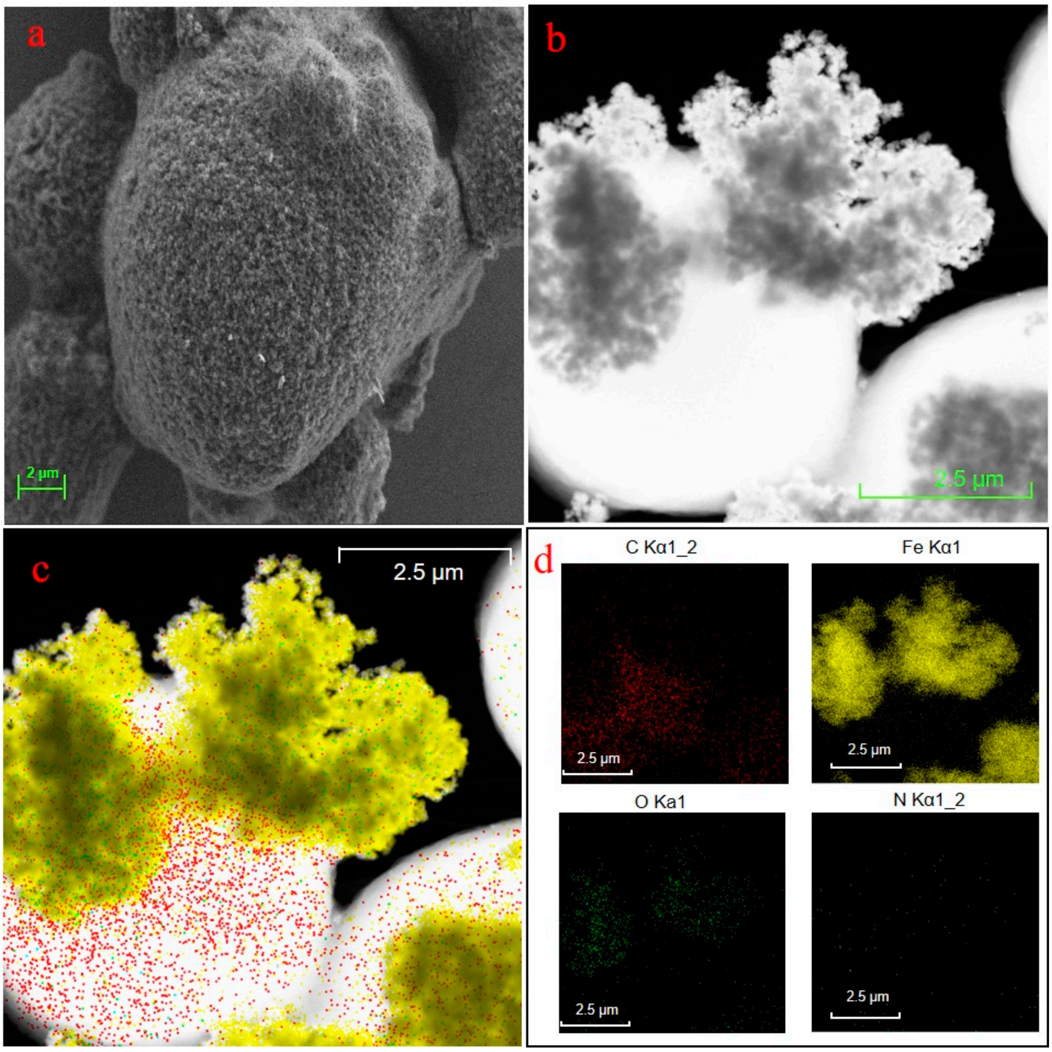

Figure S1. Morphology of MBs. (a) SEM image of MBs. (b) TEM image of MBs. (c-d) Elemental mapping of MBs.

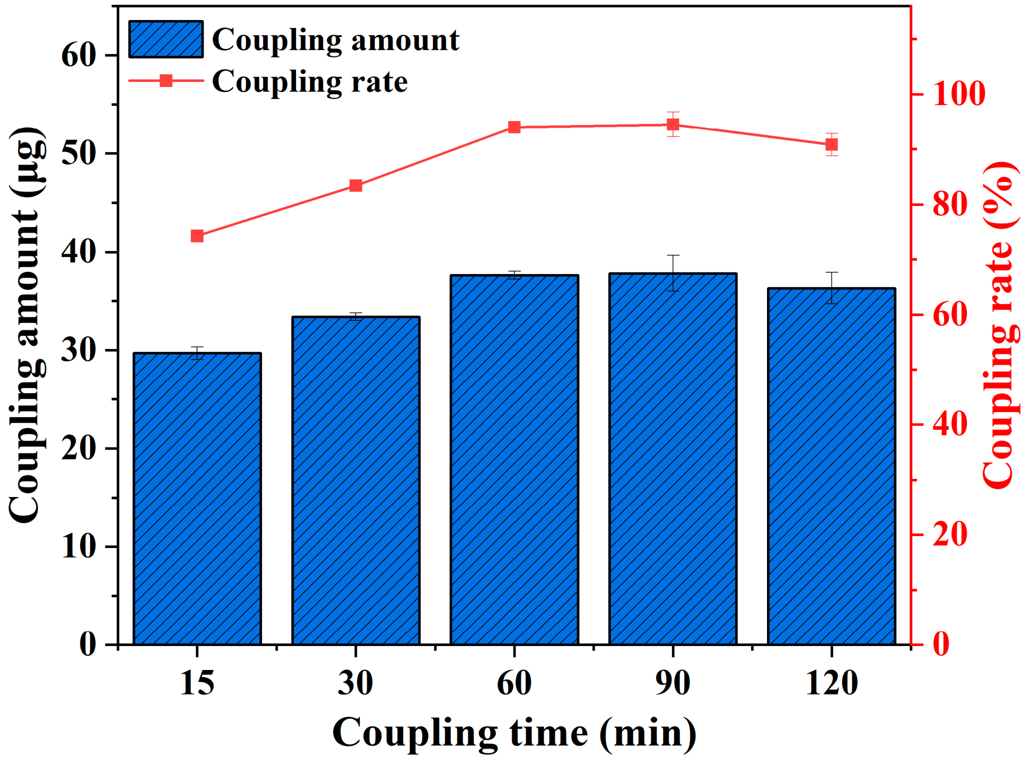

Figure S2. Coupling amount and coupling rate with different coupling times. Data were represented as mean ± standard deviation (SD) of three replicates.

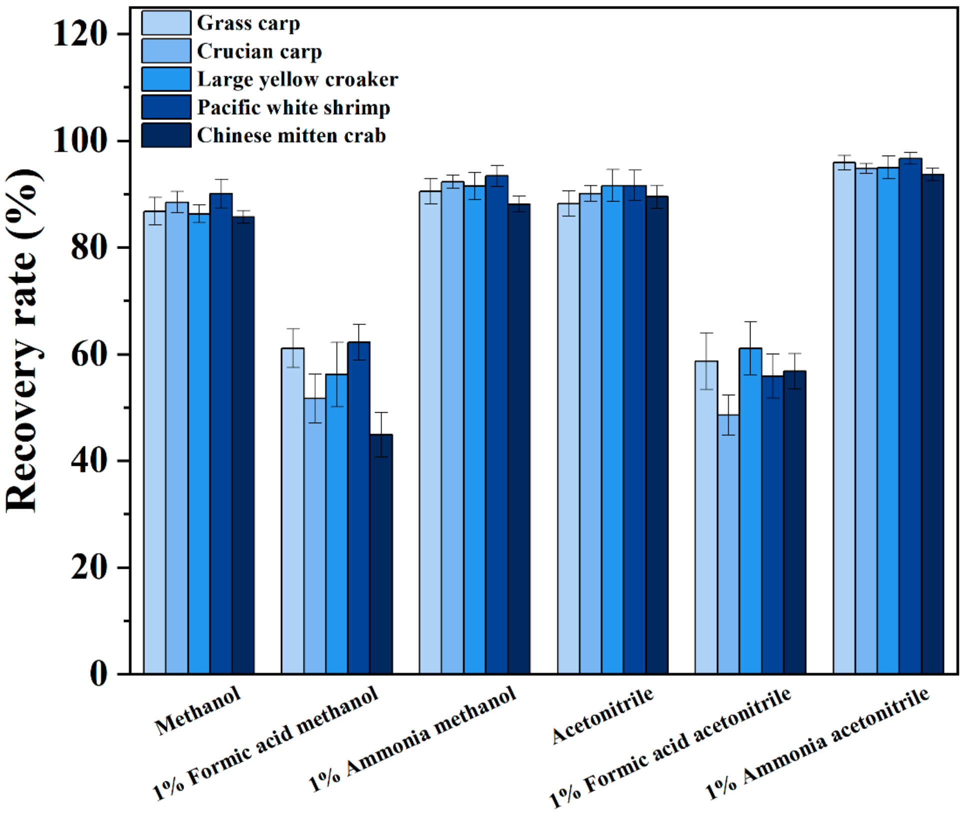

Figure S3. DZP recovery rate with different extraction solutions. Data were represented as mean ± standard deviation (SD) of three replicates.

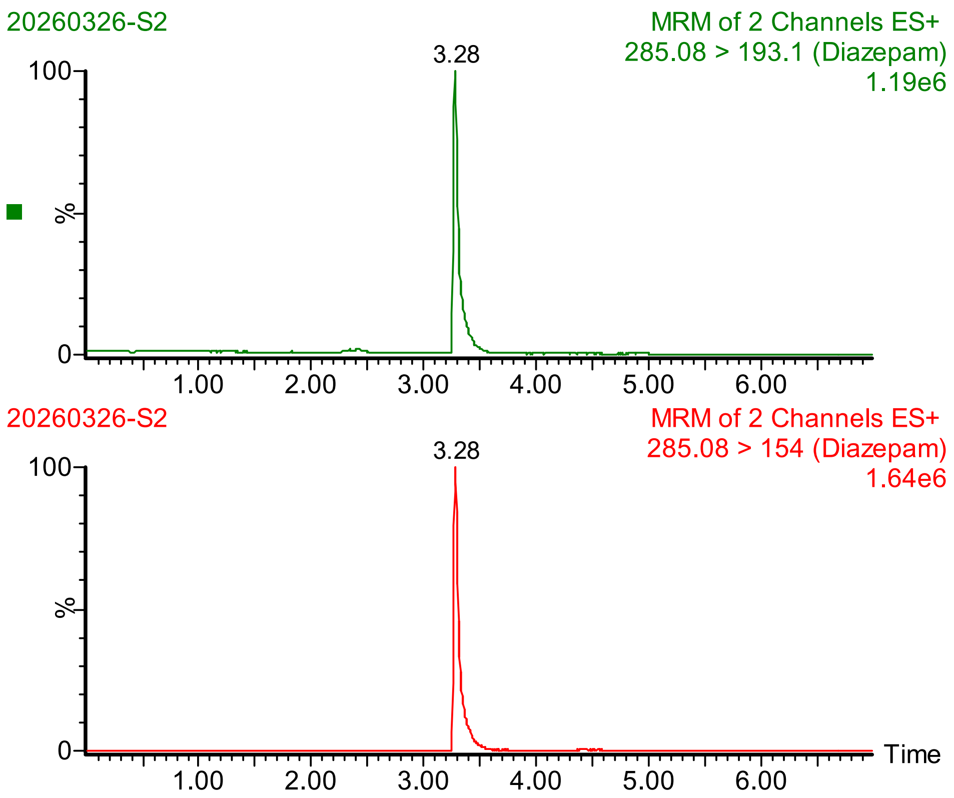

Figure S4. The MRM chromatogram of DZP standard solution (1 ng/mL).

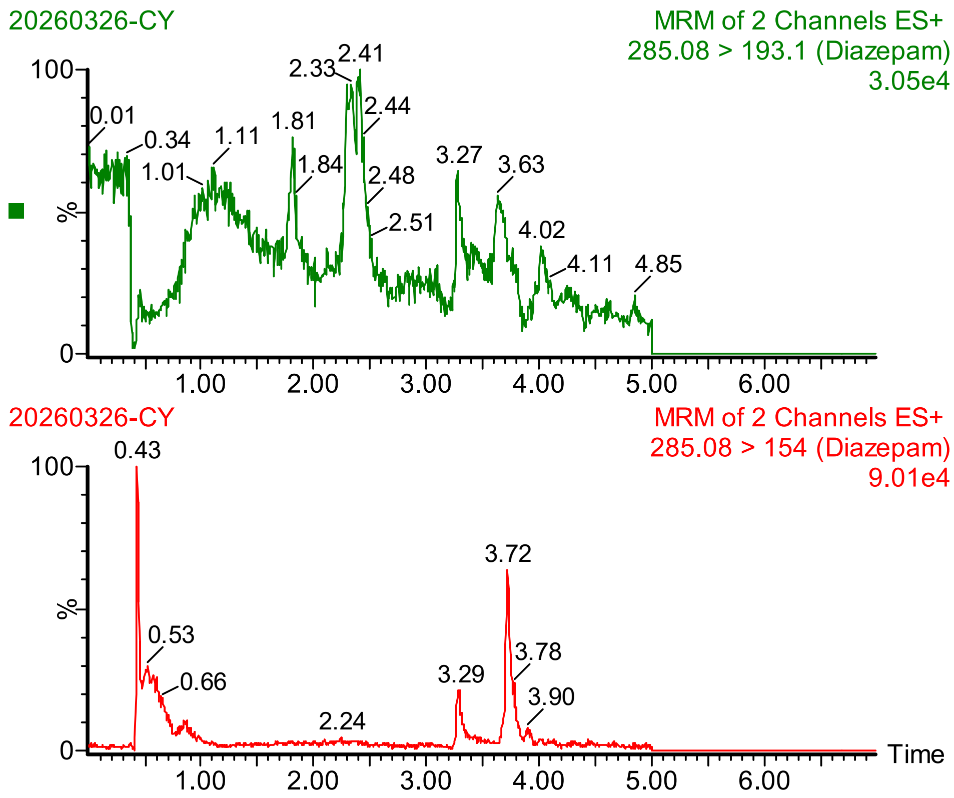

Figure S5. The MRM chromatogram of DZP in a blank grass carp matrix.

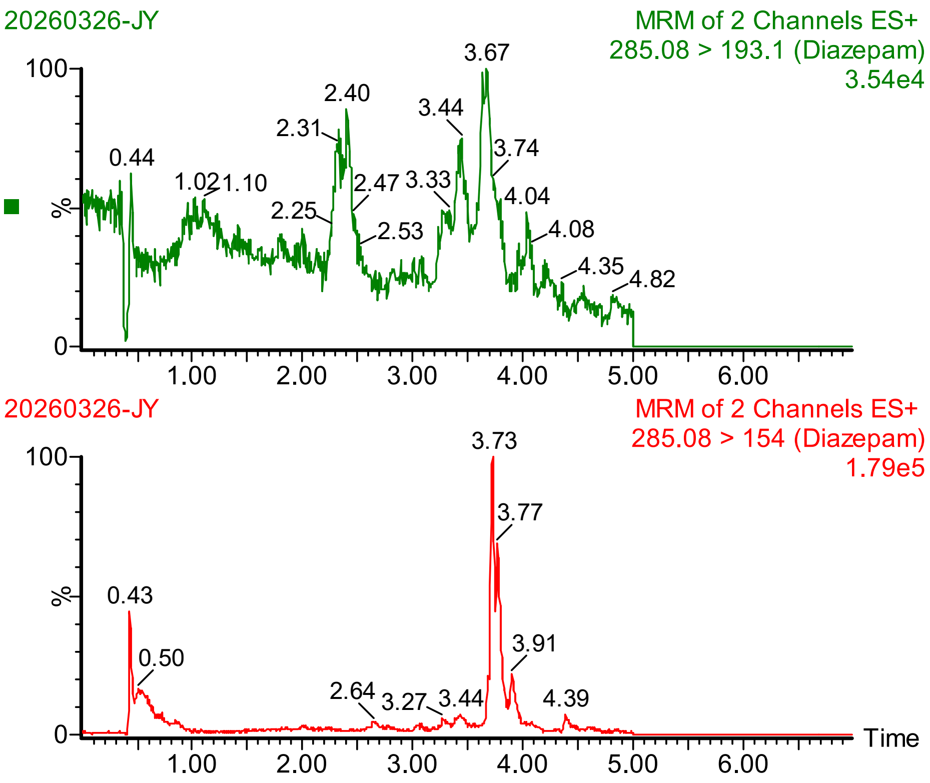

Figure S6. The MRM chromatogram of DZP in a blank crucian carp matrix.

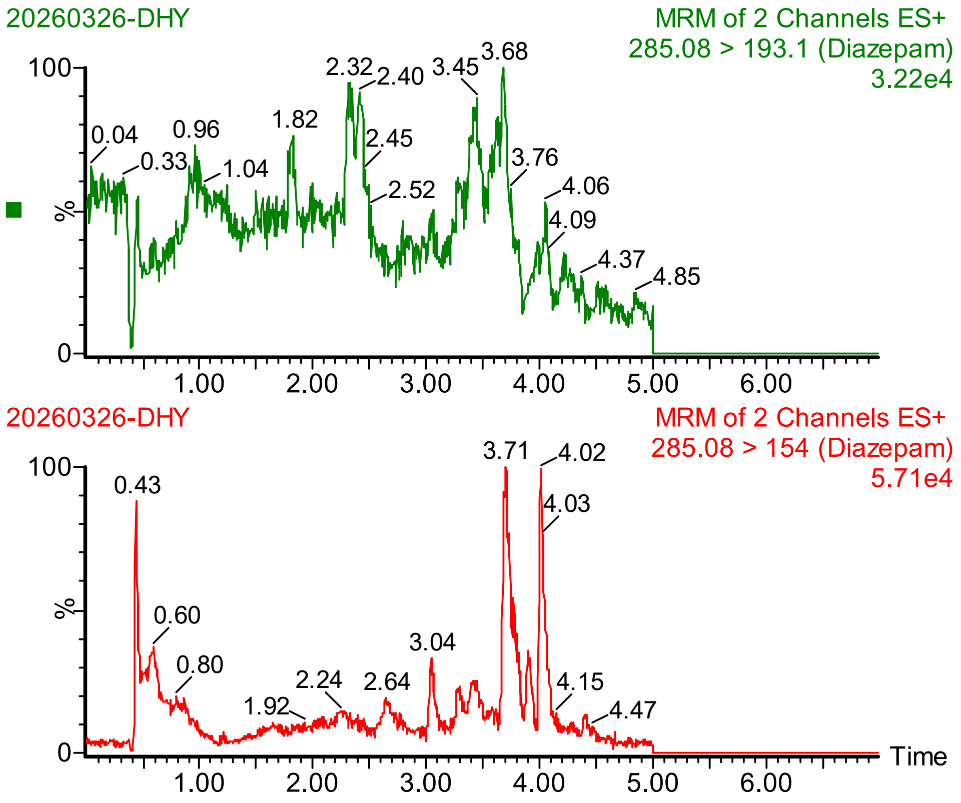

Figure S7. The MRM chromatogram of DZP in a blank large yellow croaker matrix.

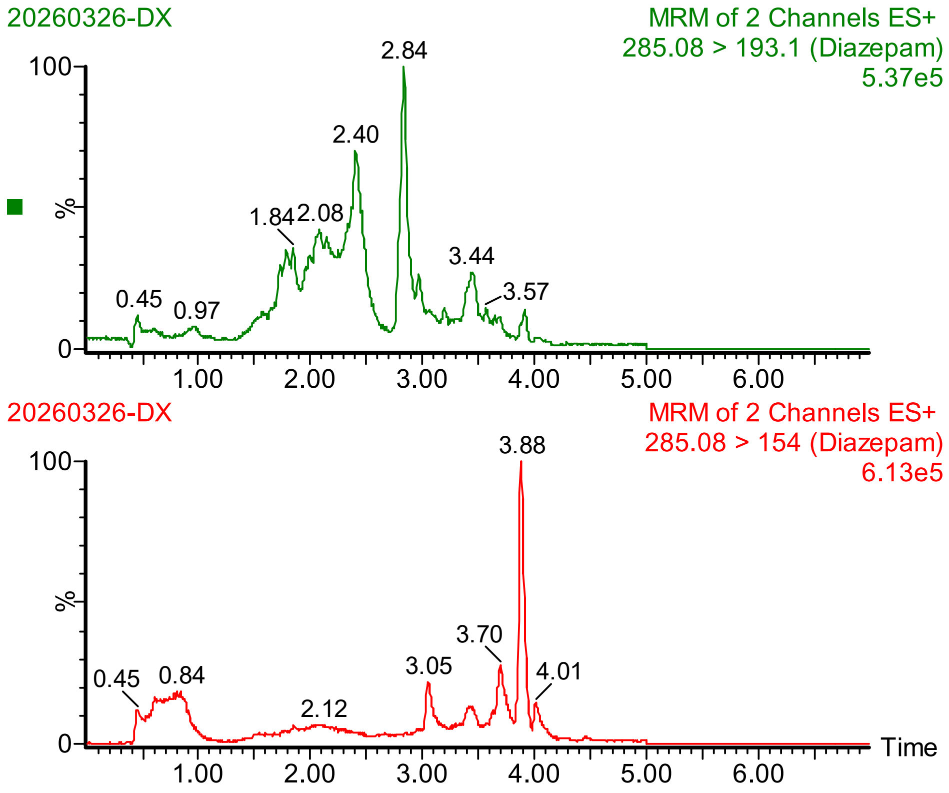

Figure S8. The MRM chromatogram of DZP in a blank Pacific white shrimp matrix.

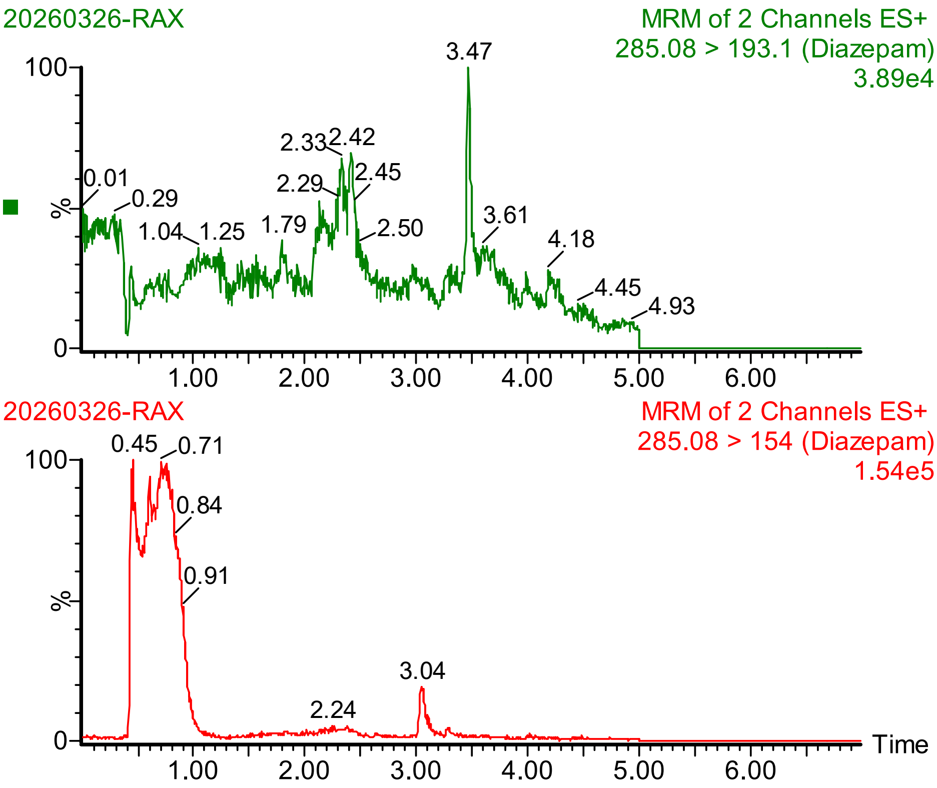

Figure S9. The MRM chromatogram of DZP in a blank Chinese mitten crab matrix.

Table S1. Atomic percentage contents of IMBs and MBs.

	C (%)	Fe (%)	O (%)	N (%)
IMBs	80.31	10.96	7.40	1.33
MBs	22.99	74.33	3.59	0.10
